# Real-world effectiveness, satisfaction, and optimization of ubrogepant for the acute treatment of migraine in combination with onabotulinumtoxinA: results from the COURAGE Study

**DOI:** 10.1186/s10194-023-01622-0

**Published:** 2023-08-03

**Authors:** Aubrey Manack Adams, Susan Hutchinson, Ella Engstrom, Nicolai D. Ayasse, Daniel Serrano, Linda Davis, Katherine Sommer, Janette Contreras-De Lama, Richard B. Lipton

**Affiliations:** 1 AbbVie, 2525 Dupont Dr, Irvine, CA 92612 USA; 2 Orange County Migraine and Headache Center, Irvine, CA USA; 3 OPEN Health, Bethesda, MD USA; 4 Kolvita Family Medical Group, Mission Viejo, CA USA; 5 Albert Einstein College of Medicine, Bronx, NY USA

**Keywords:** Ubrogepant, OnabotulinumtoxinA, Chronic migraine

## Abstract

**Background:**

Individuals using onabotulinumtoxinA as a preventive migraine treatment often use acute treatments for breakthrough attacks. Data on real-world effectiveness of the small-molecule calcitonin gene–related peptide (CGRP) receptor antagonist ubrogepant in combination with onabotulinumtoxinA are limited.

**Methods:**

COURAGE, a prospective, multiple attack, observational study, evaluated the real-world effectiveness of ubrogepant (50 or 100 mg) for acute treatment of migraine in people receiving onabotulinumtoxinA, an anti-CGRP monoclonal antibody (mAb), or both. This analysis focused only on onabotulinumtoxinA users. The Migraine Buddy app was used to identify eligible participants and track response to treated attacks. For each ubrogepant-treated attack, meaningful pain relief (MPR) and return to normal function (RNF) at 2 and 4 h post-dose over 30 days was assessed. MPR was defined as a level of relief that is meaningful to the participant, usually occurring before the pain is all gone. After 30 days, satisfaction was reported on a 7-point scale and overall acute treatment optimization was evaluated using the migraine Treatment Optimization Questionnaire-4 (mTOQ-4).

**Results:**

This analysis included 122 participants who received ubrogepant and onabotulinumtoxinA and reported on 599 ubrogepant-treated attacks. Following the first ubrogepant-treated attack, MPR was achieved in 53.3% of participants 2 h post-dose and in 76.2% of participants 4 h post-dose. RNF was achieved in 25.4% of participants 2 h post-dose and in 45.9% of participants 4 h post-dose. MPR and RNF results were similar across up to 10 ubrogepant-treated attacks. After 30 days, satisfaction with ubrogepant in combination with onabotulinumtoxinA was reported by 69.8% of participants and acute treatment optimization (defined as mTOQ-4 score ≥ 4) was achieved in 77.6%.

**Conclusions:**

In this prospective real-world effectiveness study, ubrogepant treatment in onabotulinumtoxinA users with self-identified migraine was associated with high rates of MPR and RNF at 2 and 4 h as well as satisfaction and acute treatment optimization. Although the lack of a contemporaneous control group limits causal inference, these findings demonstrate the feasibility of using a novel, app-based design to evaluate the real-world effectiveness and satisfaction of treatments.

**Graphical Abstract:**

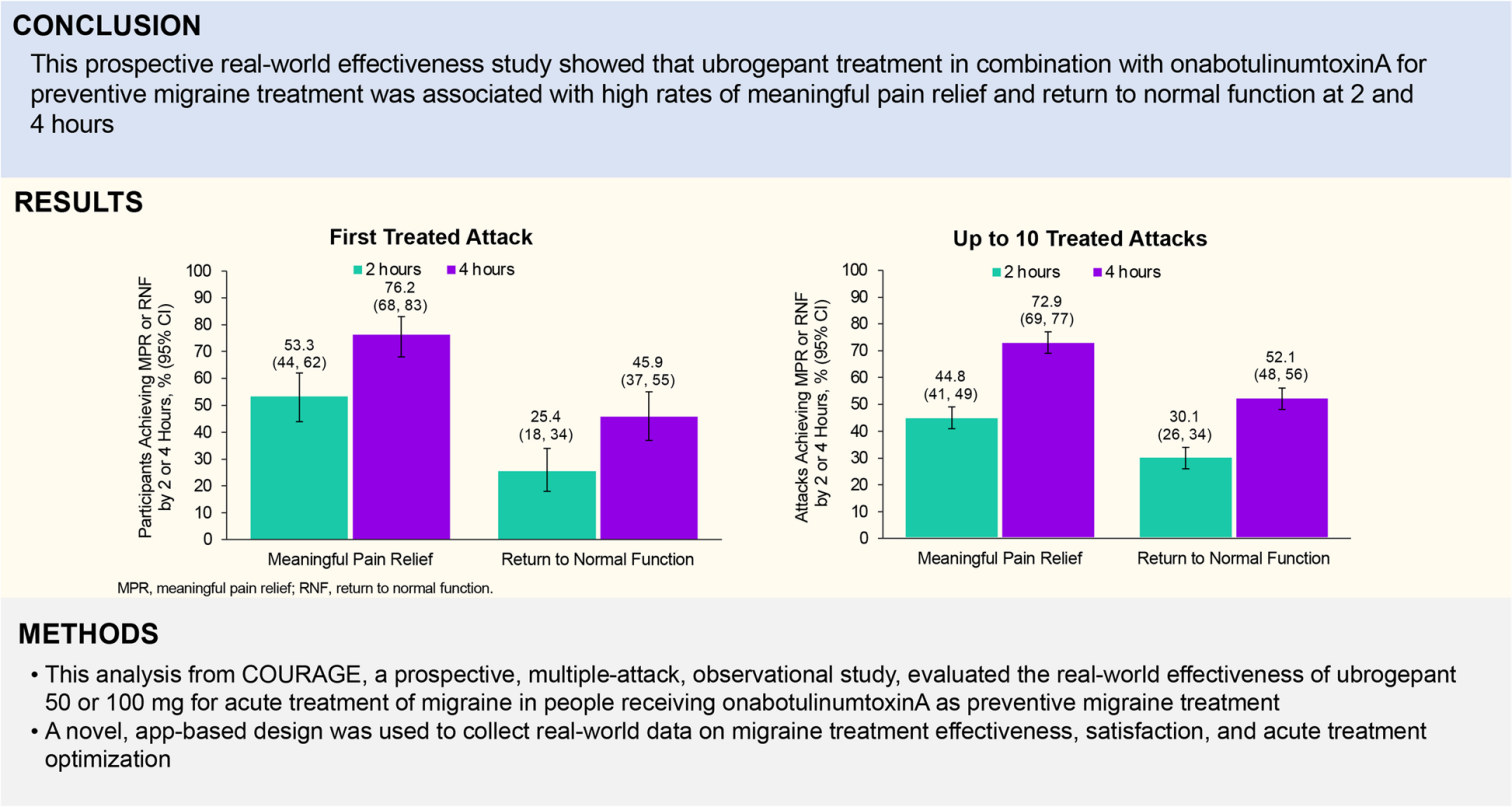

**Supplementary Information:**

The online version contains supplementary material available at 10.1186/s10194-023-01622-0.

## Introduction

The American Headache Society Consensus Statement and the European Headache Federation indicate that people on preventive treatments should be offered acute treatments for breakthrough headaches [1, 2]. Evaluating the effectiveness and safety of acute treatment in people on preventive treatments is, therefore, clinically important, particularly for individuals with chronic migraine, for whom multimodal treatment is commonly recommended [1].

The combination use of onabotulinumtoxinA, a preventive treatment for chronic migraine, and ubrogepant, an acute treatment for migraine, is of interest because of their widespread use and potentially synergistic mechanisms of action [3, 4]. Briefly, onabotulinumtoxinA works for the prevention of migraine by decreasing the release of excitatory and proinflammatory neurotransmitters and neuropeptides, including calcitonin gene–related peptide (CGRP) and glutamate from primary sensory afferents as well as reducing insertion of TRPV1 into the membranes of nociceptive neurons [5]. Ubrogepant, an approved acute treatment for migraine, also interacts with CGRP but by blocking its receptor at the onset of a migraine attack [6]. The efficacy, tolerability, and safety of ubrogepant for the acute treatment of migraine is well established in the pivotal efficacy studies and long-term safety studies [7–9]. Additionally, there is evidence that ubrogepant treatment is associated with improvement in functional outcomes, including reduced functional disability and patient satisfaction [10, 11]. While preclinical findings and clinical experience suggest that combining onabotulinumtoxinA and a small-molecule CGRP receptor antagonist may complement or even work synergistically to alleviate migraine symptoms [5], real-world data on the effectiveness of this combination are limited.

This study (COURAGE) evaluated the effectiveness of ubrogepant in combination with onabotulinumtoxinA, with an anti-CGRP monoclonal antibody (mAb), or with both onabotulinumtoxinA and an anti-CGRP mAb. The COURAGE study was conducted using Migraine Buddy (Healint, Singapore), a downloadable application (app) that helps people with migraine track and understand their migraine attacks using baseline questionnaires, customized surveys, and customizable daily diaries. Migraine Buddy was used to identify potentially eligible participants, to screen and enroll participants, to capture daily data on treated attacks, and to assess overall results at the end of a 30-day observation period.

The objective of this analysis was to evaluate the real-world effectiveness, treatment satisfaction, and acute treatment optimization of ubrogepant in combination with onabotulinumtoxinA in an open-label, 30-day, multiple-attack, observational study.

## Methods

### Study design and participants

**Fig. 1:**
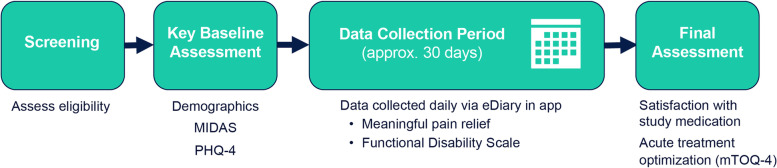
Study design. MIDAS, Migraine Disability Assessment; mTOQ-4, Migraine Treatment Optimization Questionnaire; PHQ-4, Patient Health Questionnaire-4

The real-world effectiveness of ubrogepant (50 or 100 mg) for the acute treatment of migraine when taken with onabotulinumtoxinA, a CGRP-targeted mAb, or both was evaluated in a prospective, multiple-attack, observational study (Fig. 1). Users of the Migraine Buddy app who reported using ubrogepant and onabotulinumtoxinA or anti-CGRP mAbs, or both, were potentially eligible for this study. Individuals with self-identified migraine used Migraine Buddy to record and track their migraine attacks, the characteristics of their attacks, and treatment or strategies used to manage their attacks. Following a screening questionnaire, eligible participants completed a baseline questionnaire and were enrolled in a 30-day observation period. During the observation period, participants received daily questionnaires. Effectiveness of treatment with ubrogepant was reported using an electronic diary format in the Migraine Buddy app. After 30 days, participants responded to questions about satisfaction and completed the migraine Treatment Optimization Questionnaire-4 (mTOQ-4). Three treatment arms were included in this study: ubrogepant and onabotulinumtoxinA, ubrogepant and an anti-CGRP mAb, and ubrogepant and both onabotulinumtoxinA and an anti-CGRP mAb. Here, we focus on data collected from the ubrogepant and onabotulinumtoxinA arm. The analysis of the effectiveness, satisfaction, and acute treatment optimization of the ubrogepant and an anti-CGRP mAb arm (ie, erenumab, galcanezumab, fremanezumab, or eptinezumab) will be presented in a separate publication.

Eligible participants for these analyses were Migraine Buddy users who were ≥ 18 years of age, reported ≥ 3 migraine attacks in the previous 30 days, had treated ≥ 3 prior attacks with ubrogepant (50 or 100 mg), and used onabotulinumtoxinA. Participants reported currently taking onabotulinumtoxinA to prevent migraine attacks and reported receiving onabotulinumtoxinA injections in the forehead, side and back of the head, and neck.

### Endpoints

#### Effectiveness

Effectiveness was measured by assessing meaningful pain relief (MPR) and return to normal function (RNF) at 2 and 4 h after dosing. Using the Migraine Buddy app, participants rated their headache pain level as none, mild, moderate, or severe at the time of treatment. At the end of the day, and at least 4 h since taking ubrogepant, participants were asked if they had achieved MPR. MPR was defined as a level of relief that is meaningful to the participant; participants were told that meaningful relief usually occurs before the pain is all gone. If participants treated less than 4 h before their evening diary deadline, they answered questions about their attack the next morning. If they achieved meaningful relief, they indicated how long after treatment this endpoint was achieved using the following response options: less than 1 h, 1 to 2 h, after 2 h to 4 h, and after 4 h. Prespecified outcomes were the proportion of people achieving MPR within 2 h and within 4 h of treatment. MPR is a commonly used endpoint in acute treatment for migraine and other pain disorder studies [12–14].

RNF was defined as the timepoint when the participant was fully able to function normally. The Functional Disability Scale (FDS) was used by participants to rate their ability to perform daily activities at the point when ubrogepant was taken. Responses ranged from no disability (able to function normally) to severely impaired (cannot do all or most things; bed rest may be necessary). Achieving normal function was classified as either remaining free of disability or by reporting functional disability prior to taking ubrogepant and then indicating a return to normal function at 2 and 4 h post-dose. This endpoint was rated binarily as achieving normal function or not.

#### Satisfaction and acute treatment optimization

Overall satisfaction with ubrogepant and satisfaction with ubrogepant in combination with current preventive was reported through the Migraine Buddy app at the final questionnaire using a 7-point scale ranging from “extremely dissatisfied” to “extremely satisfied.” The scale was dichotomized; satisfaction was defined by the top 3 points on the scale, “Satisfied,” “Very satisfied,” or “Extremely satisfied.” Participants reported on their overall satisfaction with ubrogepant, and satisfaction with ubrogepant in combination with their current preventive treatment.

The mTOQ is a validated and self-administered tool for people with migraine [15]. The mTOQ-4, a 4-item questionnaire that consists of frequency-based response options, was completed via the Migraine Buddy app at the final questionnaire. Sum scores range from 0 to 8, with higher scores indicating greater acute treatment optimization. Acute treatment optimization was defined by an mTOQ-4 score of at least 4. For these analyses, this outcome was rated as achieving acute treatment optimization or not.

### Statistical analysis

Continuous variables were summarized by using descriptive statistics and categorical variables were reported as frequency counts and the percentage of participants in corresponding categories. Observed data were employed for the analysis of populations. Percentages were calculated based on the number of participants with nonmissing data in each treatment group. All analyses were performed using a combination of SAS (SAS Software, Version 9.4, SAS Institute Inc, Cary, NC) and R statistical software (R, version 3.4.3, R Development Core Team).

First attack endpoints were modeled via generalized linear models parameterized with binomial distribution and logit link. The outcome event was achieving the specific endpoint, and the outcome reference was the failure to achieve the endpoint. The repeated attack diary endpoints were modeled using a generalized linear model parameterized with binomial distribution and logit link, adjusting for correlated repeated attacks via generalized estimating equations. This analysis assessed up to the first 10 treated attacks per participant because the small sample size thereafter caused convergence issues (ie, precluded the statistical model from successfully fitting to the data). Results from the unadjusted models are reported in this analysis. The reported point and interval estimates corresponded to the odds of achieving the endpoint in each model.

## Results

### Participants

Of the 134 participants enrolled in the ubrogepant and onabotulinumtoxinA arm, 12 participants were excluded from this analysis because they did not have any diary data on treated attacks. The survey study was open from September 2020 through April 2021. This analysis included 122 participants who reported at least 1 ubrogepant-treated attack while using onabotulinumtoxinA for preventive treatment of migraine (Table 1). The participants were primarily female (95.9%) and had a Migraine Disability Assessment (MIDAS) grade of IVa or IVb (86.9%). Ubrogepant 100 mg was used by 55.7% of participants and ubrogepant 50 mg was used by 44.3% of participants. A total of 599 attacks were treated with ubrogepant, with 494 attacks treated with 1 dose of ubrogepant and 105 attacks treated with 2 doses of ubrogepant. The median (interquartile range [IQR]) number of recorded attacks per participant was 9.0 (6.0;12.0) and the median (IQR) of treated attacks per participant was 5.0 (3.0;6.0).

**Table 1:** Baseline demographics

	**Ubrogepant + OnabotulinumtoxinA**^a^ ***n* = 122**
Age, mean (SD), years	40.4 (10.3)
Female, n (%)	117 (95.9)
Race, White, n (%)	107 (92.2)^b^
PHQ-4,^c^ mean (SD)	7.8 (3.0)
MIDAS grade, n (%)	
I (minimal)	3 (2.5)
II (mild)	3 (2.5)
III (moderate)	10 (8.2)
IVa (severe)	20 (16.4)
IVb (very severe)	86 (70.5)
Ubrogepant, n (%)	
50 mg	54 (44.3)
100 mg	68 (55.7)

*Abbreviations*: *MIDAS* Migraine Disability Assessment, *PHQ-4* Patient Health Questionnaire-4, *SD* standard deviation

^a^Excluding 2 missing responses, all participants reported that they received onabotulinumtoxinA injections in the forehead, side and back of head, and neck

^b^Percentage is out of the number of participants with available race data (*n*=116)

^c^PHQ-4 scale ranges from 0–12 with total scores as follows: normal (0–2), mild (3–5), moderate (6–8), severe (9–12)

### Effectiveness

**Fig. 2:**
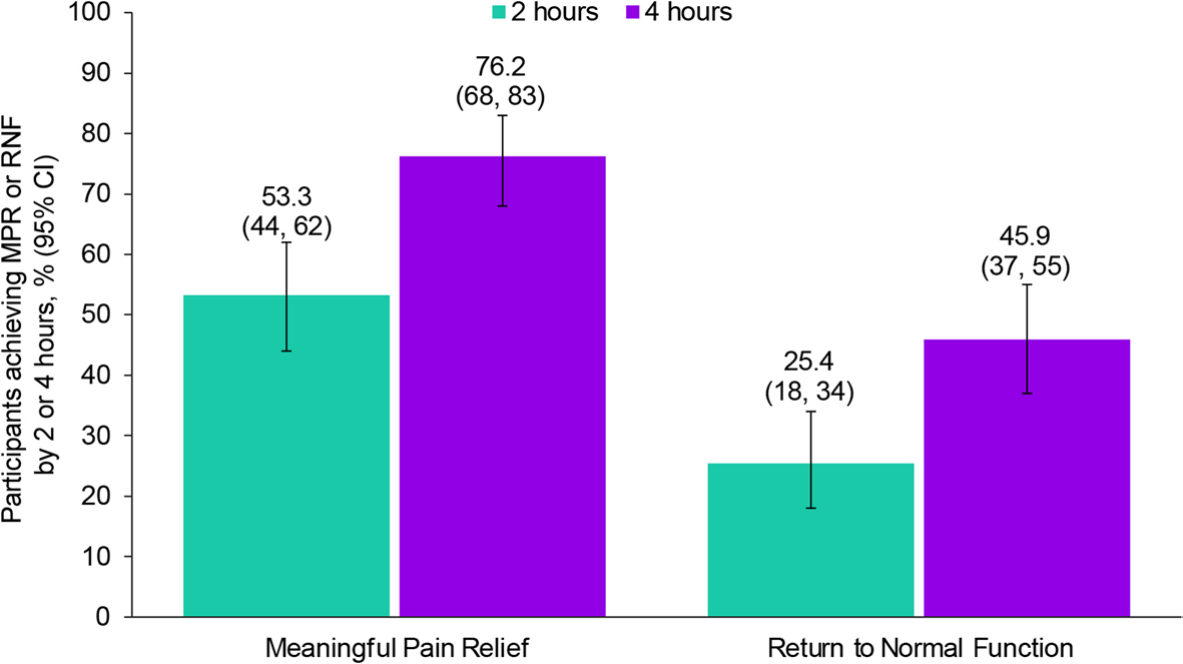
Achievement of meaningful pain relief and return to normal function for first treated attack in respondents who used ubrogepant and onabotulinumtoxinA

**Fig. 3:**
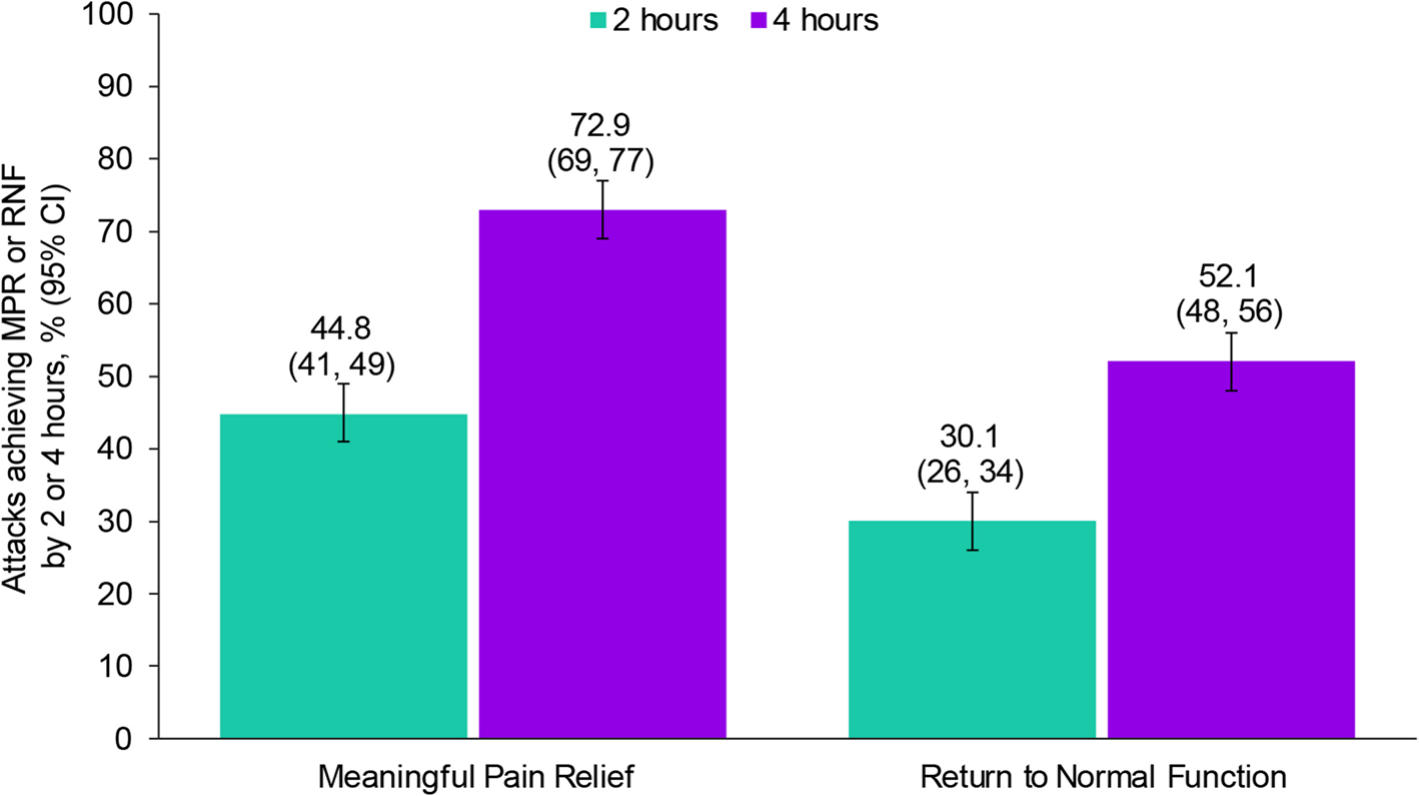
Achievement of meaningful pain relief and return to normal function across up to 10 treated attacks in respondents who used ubrogepant and onabotulinumtoxinA

MPR was achieved in 53.3% (65/122; 95% CI: 44–62%) and 76.2% (93/122; 95% CI: 68–83%) of participants at 2 and 4 h post-dose, respectively, for the first treated attack (Fig. 2); 1 participant reported a pain level of “none” pre-dose, and maintained pain-free status at 2 h post-dose. RNF was achieved by 25.4% (31/122; 95% CI: 18–34%) and 45.9% (56/122; 95% CI: 37–55%) of participants at 2 and 4 h post-dose, respectively. A pre-dose function level of no disability was reported by 5 participants who were treated; 4 of the participants maintained normal function and 1 developed functional impairment. The analysis of up to 10 ubrogepant-treated attacks included 591 attacks (Supplementary Table 1). MPR across up to 10 ubrogepant-treated attacks at 2 and 4 h post-dose was achieved by 44.8% (265/591; 95% CI: 41–49%) and 72.9% (431/591; 95% CI: 69–77%), respectively (Fig. 3). MPR remained relatively stable across up to 10 attacks (Supplementary Fig. 1). Across up to 10 ubrogepant-treated attacks, RNF was achieved by 30.1% (178/591; 95% CI: 26–34%) and 52.1% (308/591; 95% CI: 48–56%) at 2 and 4 h post-dose, respectively. Across up to 10 attacks, RNF remained relatively stable (Supplementary Fig. 2).

### Satisfaction and acute treatment optimization

**Fig. 4:**
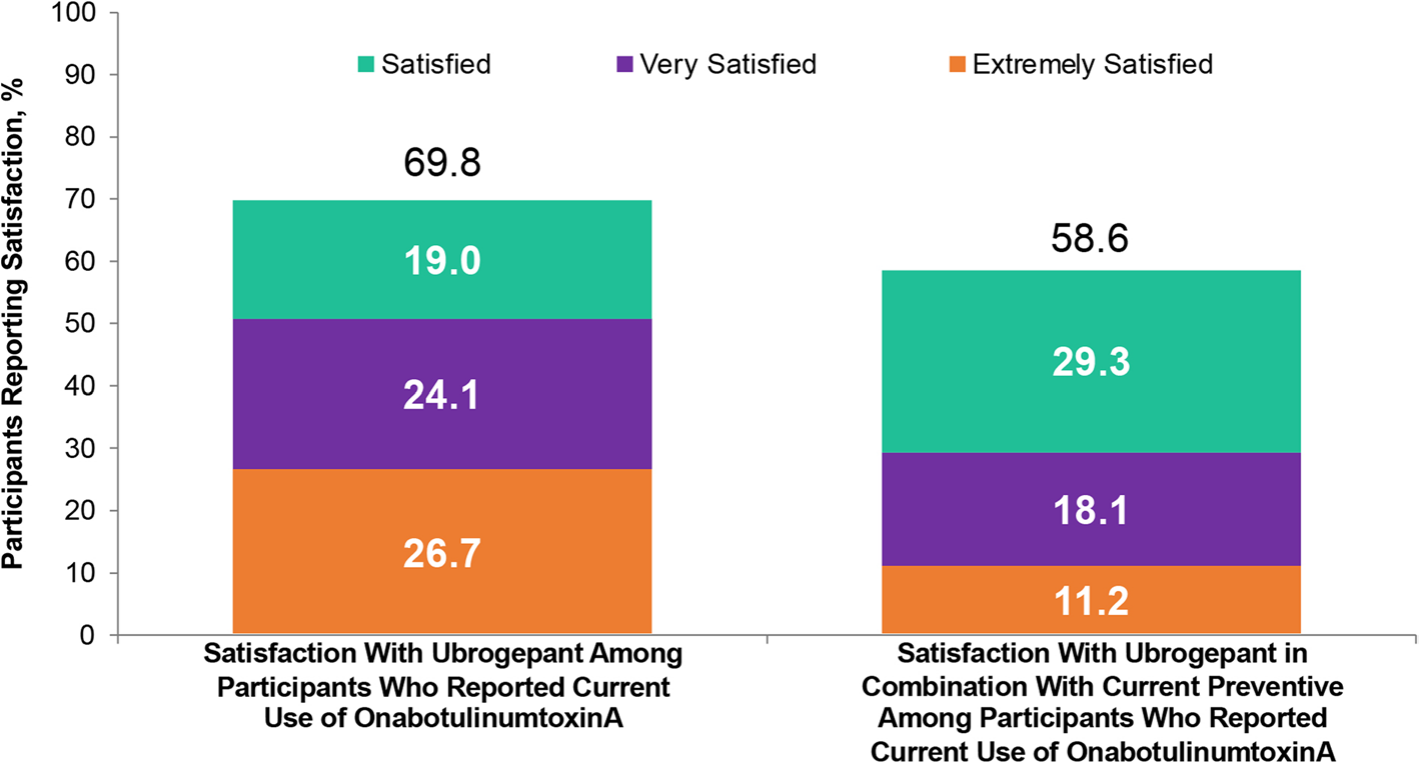
Satisfaction with ubrogepant for acute treatment of migraine and ubrogepant in combination with current preventive treatment in respondents who used ubrogepant and onabotulinumtoxinA

After 30 days of real-world use of ubrogepant with onabotulinumtoxinA, satisfaction with ubrogepant was reported by 69.8% (81/116) of participants and satisfaction with ubrogepant in combination with their current preventive was reported by 58.6% (68/116) (Fig. 4). Following the 30-day observational period, acute treatment optimization (defined as mTOQ-4 score ≥ 4) was achieved in 77.6% (90/116) of participants. The mean (standard deviation) mTOQ-4 score was 5.5 (2.6) and the median (IQR) mTOQ-4 score was 6.0 (4.0;8.0).

## Discussion

The novel, app-based design of the COURAGE study allowed collection of real-world data on migraine treatment effectiveness, satisfaction, and acute treatment optimization from the patient’s perspective. Findings suggest that ubrogepant provides relief of migraine symptoms when used to treat migraine attacks in people using onabotulinumtoxinA for preventive treatment of migraine. MPR was reported in more than 50% of participants 2 h after the first ubrogepant-treated attack. Additionally, more than 25% of participants achieved RNF at 2 h after the first treated attack. Across up to 10 ubrogepant-treated attacks, MPR and RNF remained relatively stable, which may be indicative of a consistent response with ubrogepant when used in combination with onabotulinumtoxinA. In addition, most participants reported satisfaction with ubrogepant and met the criteria for acute treatment optimization.

These findings further our understanding of ubrogepant when used with a preventive treatment. A pooled analysis from the ACHIEVE I and ACHIEVE II phase 3 and long-term safety extension trials for ubrogepant demonstrated efficacy in both participants who did and did not report preventive treatment use [16]. Additionally, the long-term use of ubrogepant in participants who were also using preventive treatments, including anticonvulsants, beta blockers, antidepressants, and onabotulinumtoxinA, was well tolerated.

Previous real-world evidence studies have demonstrated the benefits of onabotulinumtoxinA and have shown that its use is associated with decreased healthcare resource utilization [17]. Additionally, although not solely specific to the acute and preventive combination described in the present analysis, there is a growing body of evidence regarding the combination of onabotulinumtoxinA and CGRP receptor antagonists. Combination therapy may be necessary for individuals with chronic migraine who continue to experience disability after receiving a preventive treatment, and preclinical data suggest that the effect of combining onabotulinumtoxinA and a CGRP receptor antagonist may be synergistic [3]. In clinical practice, clinicians are prescribing onabotulinumtoxinA and anti-CGRP mAbs in combination, as supported by real-world data showing the effectiveness and tolerability of this combination [18, 19]. A retrospective, longitudinal chart review of adults with chronic migraine evaluating the effectiveness and tolerability of adding an anti-CGRP mAb to onabotulinumtoxinA showed that monthly headache days (MHDs) significantly decreased (mean decrease of 3.5–4.0 MHDs) over the 6–12 months of combination therapy compared with onabotulinumtoxinA alone [19]. Additionally, combination therapy was well tolerated, with a safety profile similar to each treatment alone. Similarly, a retrospective, longitudinal study that extracted data from electronic medical records found that the combination of an anti-CGRP mAb and onabotulinumtoxinA was effective and well tolerated [20]. After the addition of an anti-CGRP mAb to onabotulinumtoxinA, MHDs significantly decreased from baseline (mean decrease of 2.5–4.6 MHDs) over 3–12 months of combination therapy. Together, these findings support the effectiveness and tolerability of ubrogepant when used in combination with onabotulinumtoxinA. Future studies to evaluate compliance with ubrogepant treatment in a real-world setting are warranted [21].

Inherent limitations of observational studies exist, including the lack of a randomization process. As the objective of this real-world study was to evaluate participants who have reported treating migraine attacks with ubrogepant, this analysis also did not include a control group. Another potential limitation of this real-world study was that the collected data were self-reported, including the dose and treatment paradigm for onabotulinumtoxinA treatment. Although the data and diagnosis of migraine were not confirmed by a health care provider, participants reported use of migraine-specific products, which require a prescription from a health care provider, and completed a migraine screening assessment. Additionally, if people were misdiagnosed and this study included some individuals without migraine, it is unlikely that any diagnostic imprecision would have led to an overestimate of the treatment effect. Consistent with clinical trials on pain [22], MPR was a key endpoint used in this study. While the MPR endpoint allowed determinable results at multiple time points by assessing results at a single time point, a limitation is that it may be prone to recall bias. Pain assessments are subjective; however, MPR has been used in prior migraine studies [12, 13] and provides valuable insight into the patient’s perspective of treatment benefit [23]. Real-world studies are able to provide data on not only the effectiveness and safety of treatments, but also data regarding patient preferences [24]. Furthermore, restoration of normal function, a primary goal of acute treatments for migraine, was addressed by evaluating RNF in this study [1]. Additionally, the app-based design allowed for continuous collection of data during the COVID-19 pandemic, while maintaining the safety of the individuals with migraine and health care providers.

## Conclusion

In this prospective real-world study among participants with self-identified migraine, ubrogepant was effective when used in combination with onabotulinumtoxinA. Most participants were satisfied with ubrogepant as acute treatment and met the criteria for acute treatment optimization. These findings also demonstrate that a novel, app-based design can successfully assess real-world treatment effectiveness, satisfaction, and optimization.

### Supplementary Information

**Additional file 1:** **Supplementary Table 1.** Number of treated attacks.

**Additional file 2:** **Supplementary Figure 1. **Proportion of respondents who used ubrogepant and onabotulinumtoxinA achieving meaningful pain relief across up to 10 treated attacks.

**Additional file 3:** **Supplementary Figure 2. **Proportion of respondents who used ubrogepant and onabotulinumtoxinA achieving return to normal function across up to 10 treated attacks.

## Data Availability

AbbVie is committed to responsible data sharing regarding the clinical trials we sponsor. This includes access to anonymized, individual and trial-level data (analysis data sets), as well as other information (eg, protocols, clinical study reports, or analysis plans), as long as the trials are not part of an ongoing or planned regulatory submission. This includes requests for clinical trial data for unlicensed products and indications. These clinical trial data can be requested by any qualified researchers who engage in rigorous, independent scientific research, and will be provided following review and approval of a research proposal and Statistical Analysis Plan (SAP) and execution of a Data Sharing Agreement (DSA). Data requests can be submitted at any time after approval in the US and Europe and after acceptance of this manuscript for publication. The data will be accessible for 12 months, with possible extensions considered. For more information on the process or to submit a request, visit the following link: https://vivli.org/ourmember/abbvie/ then select “Home.”

## Abbreviations

CGRP: Calcitonin gene–related peptide
FDS: Functional Disability Scale
IQR: Interquartile range
mAb: Monoclonal antibody
MHD: Monthly headache day
MIDAS: Migraine Disability Assessment
MPR: Meaningful pain relief
mTOQ-4: Migraine Treatment Optimization Questionnaire-4
RNF: Return to normal function
