# Supplementary material for: Real-world effectiveness, satisfaction, and optimization of ubrogepant for the acute treatment of migraine in combination with onabotulinumtoxinA: results from the COURAGE Study
Source: J Headache Pain. 2023 Aug 3;24(1):102. doi: 10.1186/s10194-023-01622-0 (PMC10399003; doi:10.1186/s10194-023-01622-0)
Supplement: Supplementary file 1 — Additional file 1: Supplementary Table 1. Number of treated attacks. [file 10194_2023_1622_MOESM1_ESM.docx]

**Supplementary Table 1.** Number of Treated Attacks

| **Ubrogepant + OnabotulinumtoxinA**  **n=122** | |
| --- | --- |
| Treated Attacks | N |
| 1 | 122 |
| 2 | 110 |
| 3 | 101 |
| 4 | 87 |
| 5 | 63 |
| 6 | 43 |
| 7 | 26 |
| 8 | 19 |
| 9 | 12 |
| 10 | 8 |
