# Supplementary material for: Real-world effectiveness, satisfaction, and optimization of ubrogepant for the acute treatment of migraine in combination with onabotulinumtoxinA: results from the COURAGE Study
Source: J Headache Pain. 2023 Aug 3;24(1):102. doi: 10.1186/s10194-023-01622-0 (PMC10399003; doi:10.1186/s10194-023-01622-0)

**Supplementary Figure 1.** Proportion of respondents who used ubrogepant and onabotulinumtoxinA achieving meaningful pain relief across up to 10 treated attacks


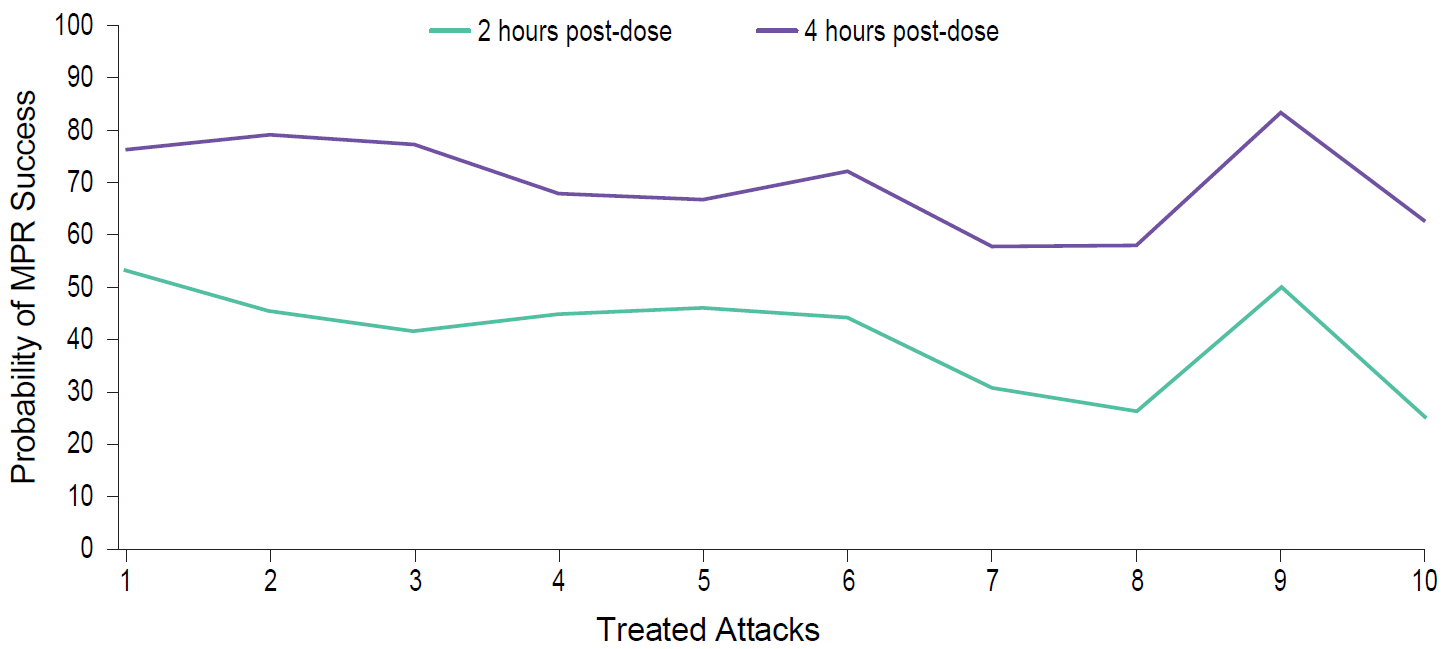

Supplement: Supplementary file 2 — Additional file 2: Supplementary Figure 1. Proportion of respondents who used ubrogepant and onabotulinumtoxinA achieving meaningful pain relief across up to 10 treated attacks. [file 10194_2023_1622_MOESM2_ESM.docx]
