# Supplementary material for: Real-world effectiveness, satisfaction, and optimization of ubrogepant for the acute treatment of migraine in combination with onabotulinumtoxinA: results from the COURAGE Study
Source: J Headache Pain. 2023 Aug 3;24(1):102. doi: 10.1186/s10194-023-01622-0 (PMC10399003; doi:10.1186/s10194-023-01622-0)

**Supplementary Figure 2.** Proportion of respondents who used ubrogepant and onabotulinumtoxinA achieving return to normal function across up to 10 treated attacks


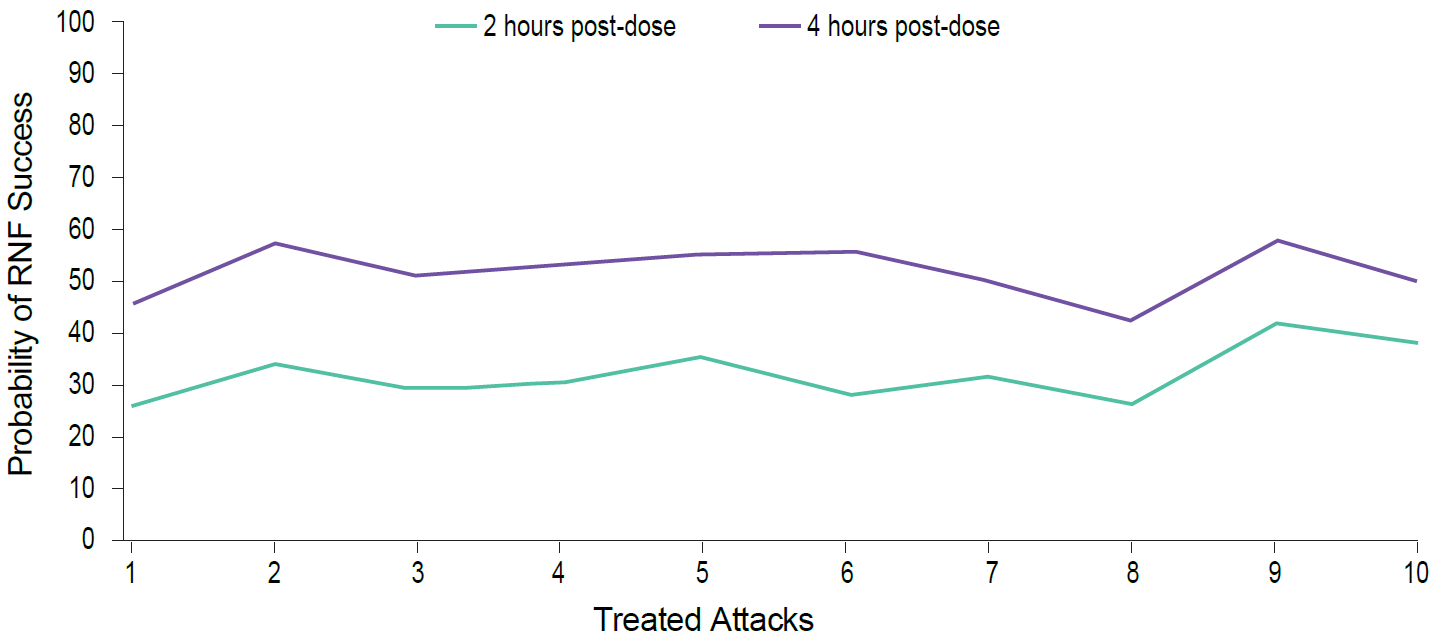

Supplement: Supplementary file 3 — Additional file 3: Supplementary Figure 2. Proportion of respondents who used ubrogepant and onabotulinumtoxinA achieving return to normal function across up to 10 treated attacks. [file 10194_2023_1622_MOESM3_ESM.docx]
